# The effectiveness of TDF versus ETV on incidence of HCC in CHB patients: a meta analysis

**DOI:** 10.1186/s12885-019-5735-9

**Published:** 2019-05-29

**Authors:** Zeyu Zhang, Yufan Zhou, Jiajin Yang, Kuan Hu, Yun Huang

**Affiliations:** Department of Hepatobiliary Surgery, Xiangya Hospital, Central South University, Changsha, Hunan China

**Keywords:** Entecavir, Tenofovir, Chronic hepatitis B, Hepatocellular carcinoma, Meta-analysis

## Abstract

**Background:**

It has been proved that nucleos(t) ide analogues (NAs) therapy could improve underlying liver disease and reduce the incidence of hepatitis B virus (HBV)-related hepatocellular carcinoma (HCC). However, the difference of effectiveness in reducing HCC occurrence between tenofovir (TDF) and enticavir (ETV), two first-line NAs drugs, is still little known. This meta analysis aims to assess the efficacy in reducing incidence of HCC comparing tenofovir monotherapy with entecavir monotherapy among chronic hepatitis B (CHB) patients by analyzing their long-term clinical outcomes.

**Methods:**

Databases including PubMed, Embase, Cochrane Central Register of Controlled Trial, and ISI Web of Science were fully investigated according to the Preferred Reporting Items for Systematic Reviews and Meta-Analyses (PRISMA) guidelines. For the included articles, two of the authors independently extracted and confirmed relevant data. Review Manager software (RevMan 5.3) was using for meta analysis.

**Results:**

Seven articles with 3698 patients were finally included in this research, 1574 in tenofovir group and 2124 in entecavir group. For meta analysis, the incidence of HCC was significantly lower among the tenofovir group than entecavir group [rate ratio (95% CI) of 0.66 (0.49, 0.89), *P* = 0.008], while there was no statistical significance in incidence of death or transplantation [rate ratio (95% CI) of 0.78 (0.55, 1.13), *P* = 0.19], encephalopathy [risk ratio (95% CI) of 0.72 (0.45, 1.13), *P* = 0.15] or variceal bleeding [risk ratio (95% CI) of 0.71 (0.34, 1.50), *P* = 0.37] between the two groups.

**Conclusion:**

There is a better effect of tenofovir in reducing HCC incidence than entecavir, which indicates tenofovir should be used more widely while treating chronic hepatitis B patients. However before applying, randomized controlled trial and large prospective cohort study should be performed in the future.

**Electronic supplementary material:**

The online version of this article (10.1186/s12885-019-5735-9) contains supplementary material, which is available to authorized users.

## Background

Chronic hepatic B virus (HBV) infection, affecting approximately 350 to 400 million people worldwide [1], is the most significant cause of liver disease that could lead to hepatocellular carcinoma (HCC) [2]. There are 15–40% of chronic HBV cases suffering from cirrhosis, while decompensate occurs one fifth of them within couple years with an essential need of transplantation [3]. HCC, a worse situation in CHB patients, is the fifth most common cancer and the third leading cause of cancer-related death in the world [4, 5]. In the established system, age, male gender, cirrhosis, positive hepatitis B e antigen (HBeAg) and high level of HBV-DNA are the risk factors for the development of HCC [4, 5]. To reduce the tremendous cost of social and economy and loss of life, effectively treating infection of HBV and preventing further HBV associated liver disease, especially HCC, are essential public health issues.

After recent decades of development in anti-HBV drugs, oral nucleos(t) ide analogues (NAs) have been recommended as the first-line therapy and widely used to inhibit HBV replication, improve underlying liver disease and reduce the incidence of HBV related HCC [6–8]. Among available NAs, entecavir (ETV) and tenofovir disoproxil fumarate (TDF) are both considered first-line regimens because of their high efficacy and low rates of resistance [8]. But the comparison between TDF and ETV remains controversial in decades. Recently, a meta analysis conducted by Zuo [9] showed that TDF was superior to ETV in suppressing HBV viral load and had a similar safety profile. However, it is still unclear about the efficacy of TDF and ETV on the HCC incidence. Since the last published systematic review in this area included only two study associated with TDF [10], several studies reporting HCC incidence in CHB patients using ETV and TDF monotherapy have been published. The purpose of our research is to compare the efficacy of TDF monotherapy with ETV monotherapy in reducing the incidence of HCC among CHB patients.

## Method

### Study selection

Two authors independently searched four databases including Pubmed, Embase, Cochrane Central Register of Controlled Trial, and ISI Web of Science following the PRISMA guidelines on August 2018. Search expressions were used as: (HBV OR hepatitis B) AND (ETV OR entecavir) AND (TDF OR tenofovir) AND (HCC OR hepatocellular carcinoma). The search details were shown in Additional file 1. Abstracts of the meeting were included and only English studies were included. Duplicated information because of overlapping patients would be excluded. In addition, we also performed a manual search of the references from the identified articles.

### Inclusion and exclusion criteria

The inclusion criteria: (1) patient population – CHB patients; (2) treatment – ETV monotherapy versus TDF monotherapy; (3) study design – randomized controlled trials, retrospective or prospective studies; (4) outcome – HCC as determined by the American Association for the Study of Liver Diseases criteria [11]. (5) total sample size > 100 and available incidence of HCC.

The exclusion criteria: (1) studies that included Human Immunodeficiency Virus or HCV co-infected patients; (2) past or present HCC or liver transplantation; (3) no patient with HCC event in either TDF or ETV group at the end of the study; (4) case(s) report.

### Data extraction

The required information was independently extracted and examined by two authors from eligible studies, including: (1) author and publication year; (2) design of study; (3) sample size; (4) basic patient information; (5) follow-up time; (6) outcomes (HCC incidence or hazard ratio (HR), death or transplantation incidence or HR, encephalopathy incidence and variceal bleeding incidence). To minimize random and bias errors in analyzing the trials, these data were extracted from the methodology sections using Cochrane methods.

### Quality assessment

The Newcastle–Ottawa Scale (The Newcastle–Ottawa Scale for assessing the quality of non-randomized studies in meta-analyses. Ottawa, Canada: Department of Epidemiology and Community Medicine, University of Ottawa.) was used to evaluated study quality [9].

### Data analysis

Review Manager software (RevMan 5.3; Cochrane Collaboration) was used for this meta-analysis. *P* < 0.05 was considered statistically significant. Data on HCC incidence, death or transplantation incidence were pooled and reported in form of events per 100 patient years of follow-up. Incidence rate ratios (IRR) and 95% confidence interval (95% CI) were pooled or calculated by method reported in previous articles [12, 13]. Rate ratios which combined IRR and HR were reported for HCC incidence and death or transplantation incidence while risk ratios (RR) with 95% CI were reported for encephalopathy incidence and variceal bleeding incidence. The heterogeneity of studies was assessed by a chi-square test and I^2^ statistics, while I^2^ > 50% was considered to be significantly heterogeneous. According to the differences of results and I^2^ of two methods, random effect method or fixed effect method was chose to combine the results of studies. Subgroup analysis and sensitivity analysis should be performed when heterogeneity was detected if necessary.

## Results

### Study selection

**Fig. 1:**
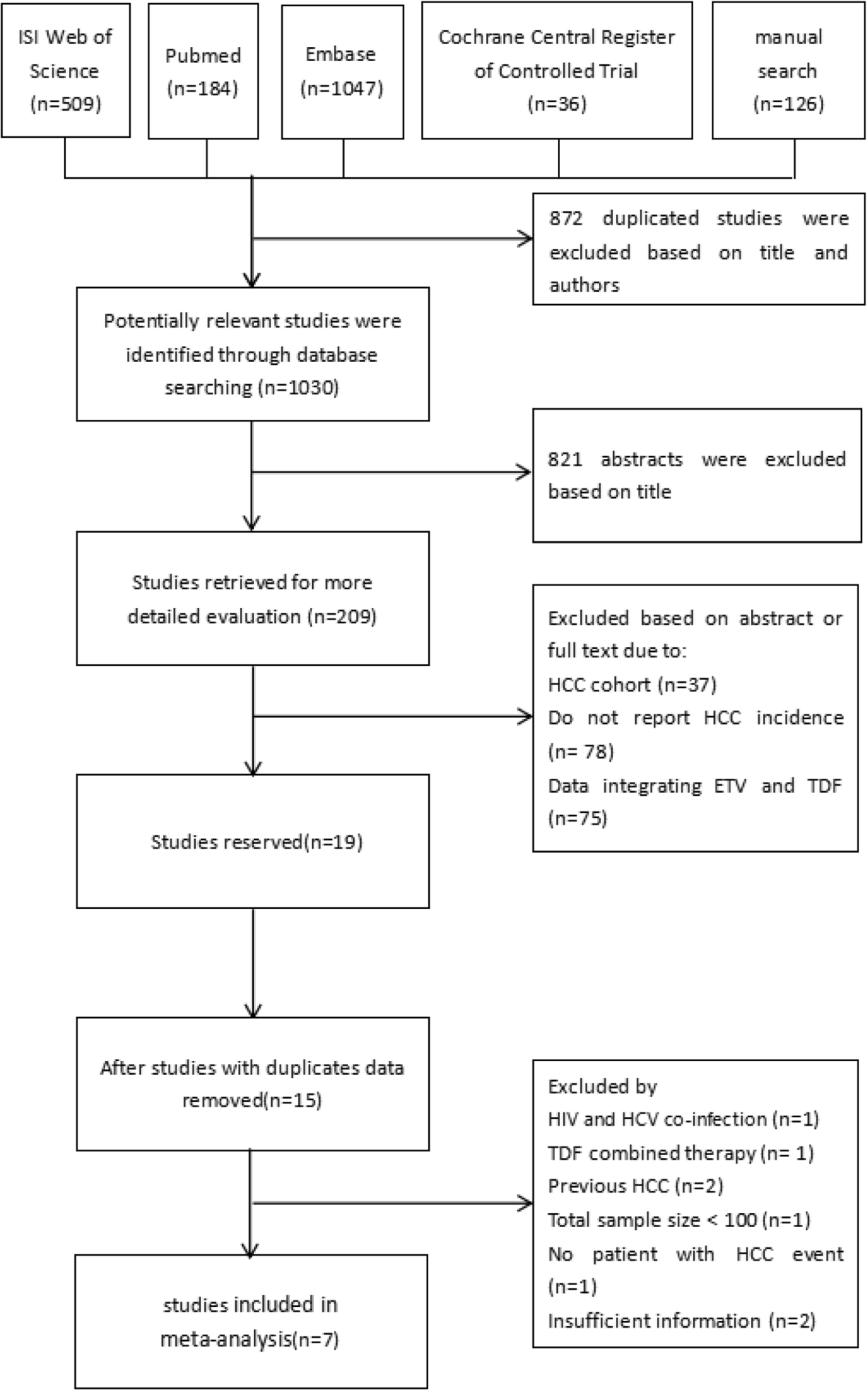
Flow diagram for the selection of studies

We initially collected 1030 articles. As showed in Fig. 1, 209 were collected for further evaluation after excluding 821 abstracts according to the title. Among them, we screened and excluded 191 studies due to: HCC cohort (*n* = 37), do not report HCC incidence (*n* = 78) and data combining ETV and TDF together (*n* = 75), by reading title, abstract or full text. In the remaining 15 unduplicated studies, a careful full text review was performed and 6 were excluded according to inclusion and exclusion criteria while two were excluded due to not providing data of event per 100 patient-year or lacking exact follow-up time causing unable to calculate IRR. Unfortunately, no RCT or real-world study was found in remaining studies. Finally, seven retrospective cohort studies with 3698 patients were included in analysis group [14–20]. It was worth mentioning that one out of seven study came from a conference abstract with a large cohort and propensity score matching [16], and our group reached the consensus to include it in this review since it provided enough information and reasonable methodology.

### Study characteristics

The studies included in the meta-analysis were summarized in Table 1. A total of 3698 CHB patients, 1574 in TDF group and 2124 in ETV group, had TDF or ETV monotherapy and the exact follow-up time for HCC occurrence in each study was shown in Table 1. All of the studies were male-dominated, and most patients were 40–60 years old with cirrhosis and detectable HBVDNA. Four of seven studies included only NAs therapy-naive CHB patients,while the rest also included non-naive. All the studies had comparable baseline data between two groups, as shown in Table 1, except patients in ETV group were older than TDF group in two studies. Besides, there was a significant difference on follow-up time between TDF and ETV group. Therefore, we used IRR instead of RR to compare the differences on HCC incidence and death or transplantation incidence.

**Table 1:** The baseline characteristics of each study

Study (Year)	Design	Sample size		Gender		Mean age (years)		N(%) HBeAg +	Liver Cirrhosis		N(%)HBVDNA detectable	NAs therapy-naive(%)		Follow-up time(m)	
		TDF	ETV	Male	Female	TDF	ETV		TDF	ETV		TDF	ETV	TDF	ETV
Koklu (2013) [14]	Retrospective cohort	72	77	114	35	54.2 ± 10.5	52.4 ± 11.2	26 (17.4)	72	77	136 (93.2)	NA	NA	21.4 ± 10.02	24.0 ± 13.18
Goyal (2015) [15]	Retrospective cohort	220	180	280	120	47.3 (24–65)	48.1 (26–65)	155 (38.7)	220	180	400 (100)	78.6	76.1	36 (11–60)	45 (12–68)
Choi (2017) [16]	Retrospective cohort	557	557	NA	NA	NA	NA	NA	557	557	NA	100	100	Up to 42	
Tsai (2017) [17]	Retrospective cohort	83	359	322	120	54.9 ± 10.9^a^	57.8 ± 10.8^a^	103 (23.3)	83	359	442 (100)	100	100	20.3 ± 6.4	43.8 ± 18.2
Kim.B.G (2018) [19]	Retrospective cohort	354	354	442	266	51 ± 11	51 ± 11	455 (64.3)	156	169	708 (100)	100	100	33 (21–46)	66 (36–88)
Kim.Y.M (2018) [18]	Retrospective cohort	112	191	186	117	49.3 ± 10.9	47.7 ± 12.3	178 (58.8)	NA	NA	303 (100)	62.5	86.4	38.5 ± 9.2	66.6 ± 26.8
Yu (2018) [20]	Retrospective cohort	176	406	376	206	49 (20–84)^a^	53 (18–84)^a^	316 (54.3)	77	148	99 (17.9)	100	100	33.6 (6.3–60.5)	69.9 (6–119.4)

*ETV* Entecavir, *TDF* Tenofovir, *SD* Standard Deviation, *NAs* nucleos(t) ide analogues, *CHB* chronic Hepatitis B, *NA* not available

^a^ with significant differences

### Quality assessment

Newcastle–Ottawa scale (NOS) was used to evaluate the quality of retrospective cohort studies, and the high quality was admitted when a NOS score > 6. The quality assessment results of seven studies were shown in Table 2. Six studies were considered as high quality but the NOS score of the only conference abstract was unclear because of lacking detail information, however it performed a propensity score matching with large cohort and comparable patient baseline data. We conjectured the NOS score was high enough to include it in this review. Besides, an updated quality assessment table (Additional file 2) subsequently proved our conjecture.

**Table 2:** The quality assessment according to the Newcastle–Ottawa quality assessment scale (NOS) of each study

	References	Koklu,(2013) [14]	Goyal(2015) [15]	Choi(2017) [16]	Tsai(2017) [17]	Kim,B.G(2018) [19]	Kim,Y.M(2018) [18]	Yu(2018) [20]
Selection	Reprensentativeness of the exposed cohort	1	1	unclear	1	1	1	1
	Selection of the non-exposed cohort	1	1	unclear	1	1	1	1
	Ascertainment of exposure	1	1	1	1	1	1	1
	Demonstration that outcome of interest was not present at the start of study	–	1	unclear	1	1	–	1
Comparibility	Study controls for age or gender	1	1	1	–	1	1	–
	Study controls for any additional factor	1	1	1	1	1	1	1
Outcome	Assessment of outcome	1	1	unclear	1	1	1	1
	Follow-up long enough for outcomes to occur	–	1	–	–	1	1	1
	Adequacy of follow-up of cohort	1	1	unclear	1	1	1	1
	Total	7	9	unclear	7	9	8	8

### Meta-analysis results

#### Incidence of HCC

**Fig. 2:**
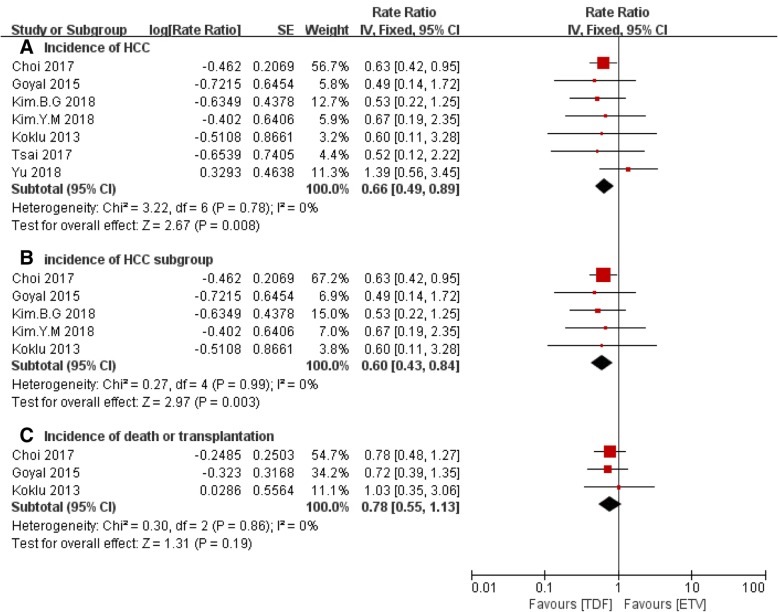
**a** Forest plot of incidence of HCC between TDF group and ETV group. **b** Forest plot of incidence of HCC between TDF group and ETV group excluding two studies with significant different age. **c**. Forest plot of incidence of death or transplantation between TDF group and ETV group

The result of HCC incidence between TDF and ETV groups was shown in Fig. 2a. In the final result, the HCC incidence was significant lower in TDF group than ETV group [rate ratio (95% CI) of 0.66 (0.49, 0.89), *P* = 0.008], though only one study reported a significant benefit in TDF group comparing to ETV group. A chi-square test and I^2^ statistics showed homogeneous data extracted from the seven studies [Chi^2^ = 3.22, degrees of freedom (df) = 6 (*P* = 0.78); *I*^2^ = 0%] and fixed effect model was used. A funnel plot analysis of publication bias was shown in Additional file 3. In order to avoid influence of age difference in incidence of HCC, we also performed a subgroup analysis excluding the two studies with significant difference of age and the result was shown in Fig. 2b. The HCC incidence was still significant lower in TDF group [rate ratio (95% CI) of 0.60 (0.43, 0.84), *P* = 0.003] with homogeneous pooled data.

#### Incidence of death or transplantation

Five studies reported incidence of death or transplantation, however there was no event in TDF group of one study [17] and in ETV group of another study [19]. Eventually three studies were included in the analysis and the results were shown in Fig. 2c. As the figure showed, there was no statistically significant difference between TDF and ETV groups using fixed effect model [rate ratio (95% CI) of 0.78 (0.55, 1.13), *P* = 0.19] without significant between-study heterogeneity [Chi^2^ = 0.30, df = 2 (*P* = 0.86); *I*^2^ = 0%].

#### Incidence of encephalopathy

**Fig. 3:**
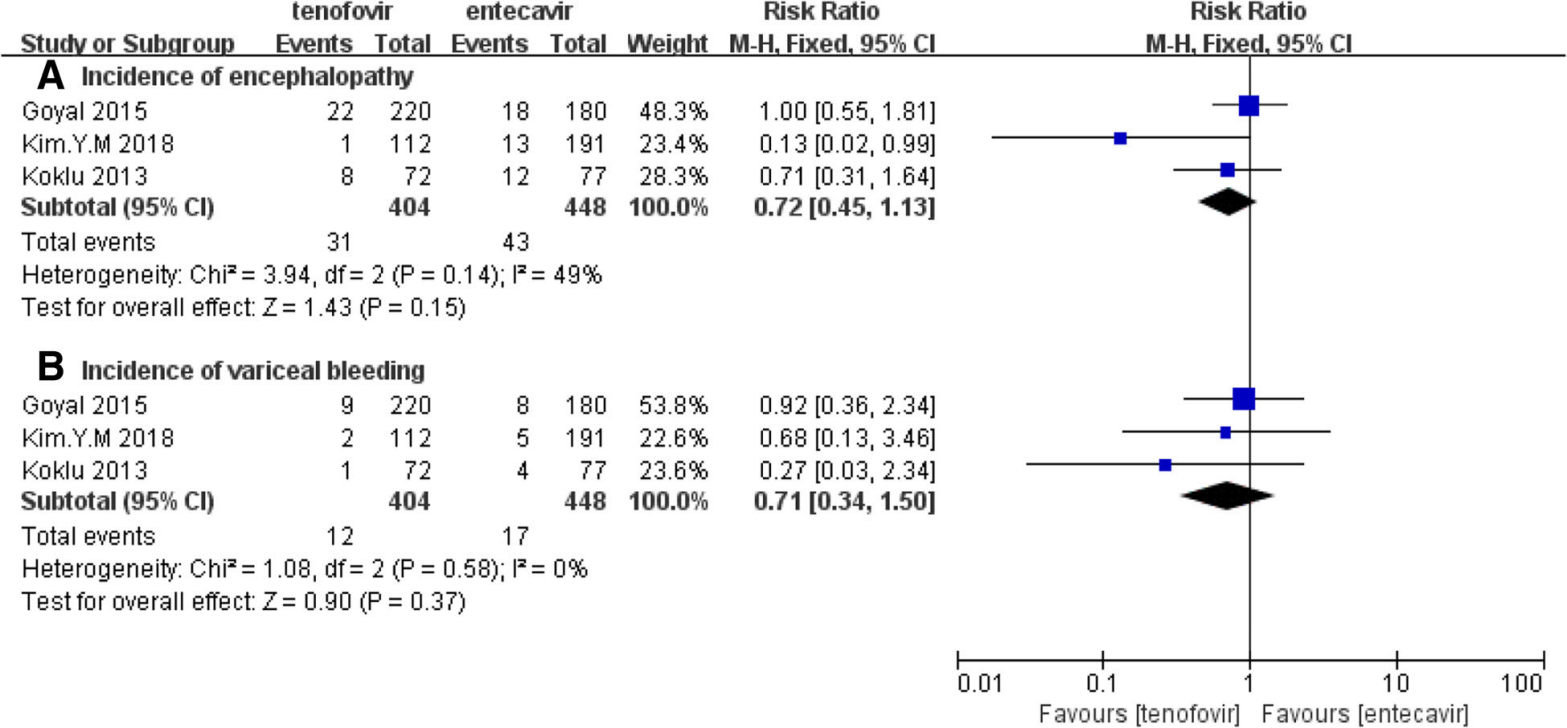
**a** Forest plot of incidence of encephalopathy between TDF group and ETV group. **b** Forest plot of incidence of variceal bleeding between TDF group and ETV group

Three studies that reported incidence of encephalopathy were included in this part of analysis with 852 patients, including 404 were tenofovir treated and 448 entecavir treated (Fig. 3a). According to the Chi^2^ and I^2^ analyses, no heterogeneity was detected between the 3 studies [Chi^2^ = 3.94, df = 2 (*P* = 0.14); *I*^2^ = 49%]. A fixed effect model was selected analyzing the data. And the difference in the incidence of encephalopathy between two drugs was insignificant [RR (95% CI) of 0.72 (0.45, 1.13), *P* = 0.15].

#### Incidence of variceal bleeding

Three studies that reported incidence of variceal bleeding were included in this part of analysis containing 404 treated with tenofovir and 448 treated with entecavir (Fig. 3b). There was no heterogeneity observed between 3 studies according to the Chi^2^ and I^2^ analyses [Chi^2^ = 1.08, df = 2 (*P* = 0.58); *I*^2^ = 0%]. A fixed effect model was used to analyze the data because of homogeneity and the difference in the number of patients that occurred event between two group was statistically insignificant [RR (95% CI) of 0.71 (0.34, 1.50), *P* = 0.37].

## Discussion

In this systematic review, seven retrospective cohort studies with 3698 patients fulfill our criteria. The current meta analysis demonstrated TDF monotherapy reduced the incidence of HCC comparing to ETV monotherapy in CHB patients with no significant differences in the incidence of death or transplantation, encephalopathy or variceal bleeding, which indicates that TDF monotherapy may be superior to ETV monotherapy in treating CHB patients.

The use of NAs, including lamivudine, telbivudine, adefovir, TDF and ETV, has been proved to be beneficial in preventing progression to cirrhosis and delaying the development of HCC in CHB patients by many research [21–24]. Meta analyses performed by Sun in 2014 [25] and Yuan in 2016 [26] both demonstrated antiviral therapy with NAs had potential benefits in reducing the recurrence rate and improving the overall survival (OS) and disease-free survival (DFS) of patients with HBV-related HCC after curative therapy. Furthermore, according to a meta analysis performed by A.K.Singal et al. in 2013 with 49 studies [26], NAs treatment significantly reduced the incidence of HCC compared with no treatment. However, the comparison of effectiveness between TDF and ETV is still little known, although it is widely accepted that these two drugs are superior to others and treated as first-line NAs drugs. In recent years, several studies have focused on comparing effectiveness of the two drugs. SR Zuo et al. performed a meta analysis comparing the efficacy of ETV and TDF with short time follow-up for the treatment of chronic hepatitis B infection [9]. Including 11 studies with 1656 patients, the results showed TDF was better able to suppress HBV viral load and had a similar safety profile as ETV. For HCC incidence, besides the studies included in the analysis, a retrospective cohort study with 346 patients by Li et al. [27], which was excluded due to lacking necessary information, showed no significant difference between TDF group and ETV group. With the same reason for excluding, a retrospective cohort study by Song et al. [28] showed higher HCC incidence in ETV than TDF arm, which is identical with our meta analysis result.

For the lower incidence of HCC in TDF group, there are several reasons that could probably explain the differences. First, recent study reveals that nucleutide analogues, rather than nucleoside analogues, provide additional effect to induce expression of interferon-λ3 [29] which will induce IFN-stimulated genes and inhibit hepatitis B surface antigen (HBsAg) production in hepatoma cells. Furthermore, interferon-λ3 has been demonstrated to be involved in modulation of immunity during virus infection or autoimmune diseases [30]. Second, TDF is related to a higher virological response rate and a lower rate of resistance comparing to ETV [31]. The uncontrolled viral status could lead to a higher risk of HCC occurrence. Lastly, the bias of the present study can not be neglected even we strictly followed the principle of PRISMA guidelines. So more studies are still needed to update this analysis. Overall, the mechanism still requires further clinical evidences.

Ascites, variceal bleeding, spontaneous bacterial peritonitis, hepatic encephalopathy and hepatorenal syndrome represent decompensation landmarks in the natural history of a cirrhotic patient, and the reported yearly rate of decompensation is 2–5% [32]. In the meantime, NAs agents are effective in restoring liver function, preventing and reversing liver decompensation, improving survival in CHB patients without significant side effects [33]. At the present study, no significant difference was found in incidence of death or transplantation, encephalopathy or variceal bleeding, and relatively few included studies (three studies) reported these data. We consider the results can be unstable with a small quantity of data. Besides, Song reported higher mortality in TDF-treated patients than ETV-treated patients [28], which is different from our result. More studies should be performed aiming at these issues. In addition, analysis of renal impairment was not performed in our study because only two of the included studies provided enough data. Nephrotoxicity with tenofovir has been demonstrated, including increases in serum creatinine and blood urea nitrogen, decrease in serum phosphate, glycosuria, proteinuria and phosphaturia in vivo experiment [34, 35]. However, real-world studies [36–39] and two of included studies [15, 17] showed controversial results. So future studies should determine the issue.

As far as we concerned, this is the first study comparing the incidence of HCC between TDF monotherapy and ETV monotherapy in CHB patients. Comparatively convincing data on all published studies are included in this study, which ensures reliable results and controls publication bias. Meanwhile, this study inspiringly show that TDF monotherapy reduces the incidence of HCC comparing to ETV monotherapy in chronic HBV patients which indicates TDF instead of ETV may be a better choice to treat CHB patients for lower incidence of HCC, thus provides relatively strong evidences for better clinical use of antiviral drugs.

There are several limitation in our meta analysis. (1) Only retrospective cohort studies met our inclusion and exclusion criteria, which mean no prospective study or RCT was included in our analysis. (2) There was still a need of large-scale studies. (3) We did not distinguish the genotype of HBV and baseline HBVDNA because of lacking adequate studies, which might result in unavoidable bias. (4) The follow-up time in TDF group was shorter than in ETV group in most of included studies, which could prevent from detecting more occurrence of HCC. Although we used event per 100 patient-year and IRR to diminish the affection to result, the bias was still unavoidable. In the mean time, longer follow-up time was needed to provide more accurate HCC incidence data. (5) Most of included studies were the result of Asian CHB cohort, which might cause bias while considering all races. An update meta-analysis should be performed in future using more comprehensive data. (6) ETV group was older than TDF group in two included studies. Although we performed a subgroup analysis to avoid bias, it still existed in some extent because the patients with older age were more likely to have HCC especially if infected at birth or early in life. (7) Different indications were applied in the included studies, making it impossible to figure out which kind of patients could benefit more from TDF therapy. (8) Some data whose exact values were unable to obtain when processing because of lacking original data.

## Conclusion

In summary, despite these limitation listed above, our study still demonstrates better effect of TDF in reducing HCC incidence than ETV, which indicates TDF to be a better choice while treating CHB patients. However, RCT and large prospective cohort study should be performed before applying.

## Additional files

Additional file 1: Search details. The search details we used in PubMed and Embase. (DOC 12 kb)

Additional file 2: Updated quality assessment table. The quality assessment according to the Newcastle–Ottawa quality assessment scale (NOS) of each study. (updated from newest published article of the Choi’s study in 2019) (DOCX 17 kb)

Additional file 3: Funnel plot analysis of publication bias. Publication bias among studies involving in the outcome impact of TDF and ETV on the incidence of HCC. (DOC 17 kb)

## Data Availability

All data generated or analysed during this study are included in this published article [and its supplementary information files].

## Abbreviations

CHB: Chronic hepatitis B
DFS: Disease-free survival
ETV: Enticavir
HBeAg: Hepatitis B e antigen
HBsAg: Hepatitis B surface antigen
HBV: Hepatitis B virus
HCC: Hepatocellular carcinoma
HR: Hazard ratio
IRR: Incidence rate ratios
NAs: Nucleos(t) ide analogues
NOS: Newcastle–Ottawa scal
OS: Overall survival
PRISMA: The Preferred Reporting Items for Systematic Reviews and Meta-Analyses
RR: Risk ratios
TDF: Tenofovir disoproxil fumarate
