# Supplementary material for: The effectiveness of TDF versus ETV on incidence of HCC in CHB patients: a meta analysis
Source: BMC Cancer. 2019 May 29;19:511. doi: 10.1186/s12885-019-5735-9 (PMC6542001; doi:10.1186/s12885-019-5735-9)
Supplement: Supplementary file 1 — Search details. The search details we used in PubMed and Embase. (DOC 12 kb) [file 12885_2019_5735_MOESM1_ESM.doc]

Pubmed:

(((HCC[All Fields] OR ("carcinoma, hepatocellular"[MeSH Terms] OR ("carcinoma"[All Fields] AND "hepatocellular"[All Fields]) OR "hepatocellular carcinoma"[All Fields] OR ("hepatocellular"[All Fields] AND "carcinoma"[All Fields]))) AND (TDF[All Fields] OR ("tenofovir"[MeSH Terms] OR "tenofovir"[All Fields]))) AND (ETV[All Fields] OR ("entecavir"[Supplementary Concept] OR "entecavir"[All Fields]))) AND (HBV[All Fields] OR ("hepatitis b"[MeSH Terms] OR "hepatitis b"[All Fields])) AND ("0001/01/01"[Date - Publication] : "2018/08/14"[Date - Publication])

Embase:

('hepatocellular carcinoma'/exp OR 'hepatocellular carcinoma' OR (hepatocellular AND ('carcinoma'/exp OR carcinoma)) OR hcc) AND ('hepatitis b'/exp OR 'hepatitis b' OR (('hepatitis'/exp OR hepatitis) AND b) OR 'hbv'/exp OR hbv) AND ('entecavir'/exp OR entecavir OR etv) AND ('tenofovir'/exp OR tenofovir OR tdf) AND [1-1-0001]/sd NOT [2-8-2018]/sd
