# Supplementary material for: The effectiveness of TDF versus ETV on incidence of HCC in CHB patients: a meta analysis
Source: BMC Cancer. 2019 May 29;19:511. doi: 10.1186/s12885-019-5735-9 (PMC6542001; doi:10.1186/s12885-019-5735-9)
Supplement: Supplementary file 2 — Updated quality assessment table. The quality assessment according to the Newcastle–Ottawa quality assessment scale (NOS) of each study. (updated from newest published article of the Choi’s study in 2019) (DOCX 17 kb) [file 12885_2019_5735_MOESM2_ESM.docx]

Updated quality assessment table. The quality assessment according to the Newcastle–Ottawa quality assessment scale (NOS) of each study. (updated from newest published article of the Choi’s study in 2019^1^)

|  | References | Koklu,(2013) | Goyal(2015) | Choi(2019) | Tsai(2017) | Kim,B.G(2018) | Kim,Y.M(2018) | Yu(2018) |
| --- | --- | --- | --- | --- | --- | --- | --- | --- |
| Selection | Reprensentativeness of the exposed cohort | 1 | 1 | 1 | 1 | 1 | 1 | 1 |
|  | Selection of the non-exposed cohort | 1 | 1 | 1 | 1 | 1 | 1 | 1 |
|  | Ascertainment of exposure | 1 | 1 | 1 | 1 | 1 | 1 | 1 |
|  | Demonstration that outcome of interest was not present at the start of study | - | 1 | 1 | 1 | 1 | - | 1 |
| Comparibility | Study controls for age or gender | 1 | 1 | 1 | - | 1 | 1 | - |
|  | Study controls for any additional factor | 1 | 1 | 1 | 1 | 1 | 1 | 1 |
| Outcome | Assessment of outcome | 1 | 1 | 1 | 1 | 1 | 1 | 1 |
|  | Follow-up long enough for outcomes to occur | - | 1 | - | - | 1 | 1 | 1 |
|  | Adequacy of follow-up of cohort | 1 | 1 | 1 | 1 | 1 | 1 | 1 |
|  | Total | 7 | 9 | 8 | 7 | 9 | 8 | 8 |

1. Choi J, Kim HJ, Lee J, Cho S, Ko MJ, Lim Y. Risk of Hepatocellular Carcinoma in Patients Treated With Entecavir vs Tenofovir for Chronic Hepatitis B: A Korean Nationwide Cohort Study. JAMA Oncol. 2019;5(1):30–36. doi:10.1001/jamaoncol.2018.4070
