# Supplementary material for: The effectiveness of TDF versus ETV on incidence of HCC in CHB patients: a meta analysis
Source: BMC Cancer. 2019 May 29;19:511. doi: 10.1186/s12885-019-5735-9 (PMC6542001; doi:10.1186/s12885-019-5735-9)
Supplement: Supplementary file 3 — Funnel plot analysis of publication bias. Publication bias among studies involving in the outcome impact of TDF and ETV on the incidence of HCC. (DOC 17 kb) [file 12885_2019_5735_MOESM3_ESM.doc]

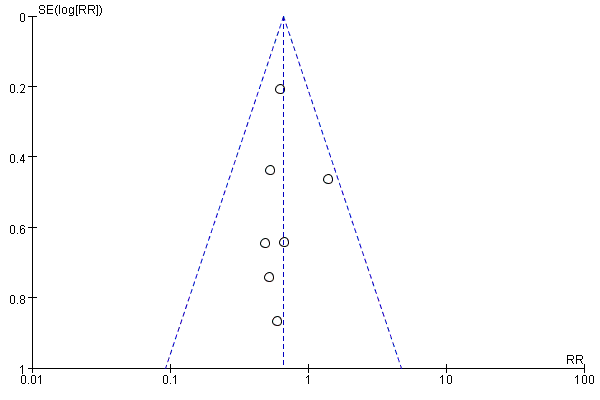


Funnel plot analysis of publication bias. Publication bias among studies involving in the outcome impact of TDF and ETV on the incidence of HCC.
